# Colonic Abnormalities in Manitoban Children with *Helicobacter pylori* Gastritis

**DOI:** 10.1155/2018/6840390

**Published:** 2018-04-02

**Authors:** Upama Banik, Camelia Stefanovici, Jennifer Griffin, Wael El-Matary

**Affiliations:** Section of Pediatric Gastroenterology, Departments of Pediatrics and Pathology, College of Medicine, Faculty of Health Sciences, University of Manitoba, Winnipeg, MB, Canada

## Abstract

**Objectives:**

Association between *Helicobacter pylori (H. pylori)* and colonic pathology is underinvestigated. The aim of this work was to examine the prevalence and nature of colonic changes in children diagnosed with *H. pylori* gastritis.

**Methods:**

A comprehensive retrospective review of the medical records for all Manitoban children (≤17 years) diagnosed with *H. pylori* gastritis from January 1996 to May 2015 was conducted. Children with *H. pylori* gastritis who had colonoscopy were identified. Patients' demographics, indications for colonoscopy, laboratory and endoscopic findings, and colonic histopathological abnormalities were documented.

**Results:**

A total of 231 children were found to have *H. pylori* gastritis. The mean age at diagnosis was 12.3 ± 4.1 years; 108 (46.6%) were girls. Of the 231 patients, 37 (16%) patients were found to have colonoscopy performed. Indications for colonoscopy included bleeding per rectum, significant weight loss, and hypoalbuminemia. Twenty-two (59%) of 37 children who had colonoscopy had significant endoscopic and histopathological findings on colonoscopy including polyposis and colitis. Boys with colonic changes were diagnosed at an earlier age compared to those without (11.5 ± 7.0 versus 15.0 ± 2.0, *p* < 0.049).

**Conclusions:**

Our study may suggest a possible association between *H. pylori* and a subset of colonic changes in children.

## 1. Introduction

*Helicobacter pylori (H. pylori)* is one of the most common bacterial infections worldwide. Approximately half of the world's population has *H. pylori* gastritis. The prevalence of *H. pylori* is not universally constant. The variability can be accounted for by the patient's age, ethnic background, and socioeconomic status [1]. The infection is more predominant and acquired at a younger age in developing nations compared with industrialized nations [2]. The prevalence among the Canadian adult population is 20–30% [3] and 7.1% in Canadian children [4]. *H. pylori* in the Canadian pediatric population is more common in Aboriginal population and first-generation immigrants [5]. In the First Nations community of Wasagamack, Manitoba, the prevalence in children from ages 6 months to 12 years was found to be as high as 67% [6].

The pathophysiology of the infection is a multifarious dynamic interaction between the host and pathogen. Prolonged infection with this pathogen stimulates chronic inflammation in gastric mucosa. Chronic inflammation in combination with altered gastric secretion and tissue injury can lead to chronic gastritis and ulcers and eventually, if left untreated, may lead to gastric cancer and MALT lymphoma. The pathogen has also been strongly associated with duodenal ulcers. *H. pylori* infection may be linked to extragastrointestinal (GI) pathology including cardiovascular disease, diabetes mellitus, lung cancer, hepatobiliary diseases, and neurological disorders such as Alzheimer's disease in adults [7]. The infection has been associated with extragastrointestinal manifestations of sideroblastic anemia and chronic idiopathic thrombocytopenic purpura in the pediatric population [8].

Although the majority of children who have *H. pylori* infection are asymptomatic, the infection persists for a lifetime unless a treatment regime is delivered. Symptomatic children may present with nonspecific indicators such as postprandial epigastric pain, nocturnal awakening, unexplained nausea and/or vomiting, anorexia, hematemesis, and iron deficiency anemia [8]. Associations have also been made between the pathogen and growth impediments [9].

Recently, we anecdotally noticed significant colonic changes in several children with *H. pylori* gastritis. Although there are several studies which look at the correlation of *H. pylori* and colonic changes in adults [10–19], there is a paucity of studies in the pediatric population. The aim of this study was to determine the prevalence of colonic changes in children with *H. pylori* and to characterize the colonic changes found.

## 2. Materials and Methods

A comprehensive retrospective chart review was performed for all children (age ≤ 17 years) diagnosed with *H. pylori* gastritis from January 1996 to May 2015 at the Children's Hospital, Winnipeg, Manitoba, Canada. Patients' demographics were obtained from pathology and pediatric gastroenterology databases.

Patients were considered to have *H. pylori* gastritis if they had one or more of the following:

1. Positive histopathology for *H. pylori*
2. Positive *Campylobacter*-like organism (CLO) test on the gastric biopsy
3. Positive gastric biopsy culture for *H. pylori*
4. Positive urea breath test for *H. pylori*

A search query was performed in the Department of Pathology's Laboratory Information System to determine which of the patients with *H. pylori* who had an ileocolonoscopy in association with gastroscopy at the time of *H. pylori* diagnosis. Gastroscopy and ileocolonoscopy findings were documented. Biopsy specimens' sites included lower esophagus, gastric body, gastric antrum, duodenal bulb and second part of duodenum and body of stomach, and esophagus. Ileocolonoscopy biopsy specimens were obtained from terminal ileum, cecum, ascending colon, transverse colon, descending colon, sigmoid colon, and rectum. Two biopsy specimens were obtained from each site and gastroscopy and colonoscopy. The biopsy specimens were processed and examined as per the general protocol for gastrointestinal specimens, including the routine hematoxylin and eosin (H&E) stain. In addition, a mandatory staining using Warthin-Starry stain for all the specimens from the gastric antrum was performed.

### 2.1. Inclusion Criteria

All children aged 17 years or less with proven *H. pylori* gastritis using the above definition and who had ileocolonoscopy for any reason during the study period were included.

Patients were excluded if they were older than 17 years of age or had gastritis that was not due to *H. pylori.*

Patient details including presenting symptoms, laboratory findings, methods of diagnosis, pathological findings, treatment, and treatment outcomes were documented. Information pertaining to the patient's height percentile, weight percentile, and BMI percentile at diagnosis were collected. Any associated chronic medical comorbid conditions or other medications that the patients were taking at the time of the diagnosis were documented. In addition, family history of any GI conditions including *H. pylori* infection was recorded. Laboratory findings including hemoglobin, erythrocyte sedimentation rate (ESR), C-reactive protein (CRP), albumin, total iron-binding capacity (TIBC), serum ferritin, and iron levels were documented at the time of diagnosis. The following definitions for laboratory values, as defined by Diagnostic Services Manitoba, were used to classify laboratory values as normal versus abnormal:

1. Low hemoglobin: hemoglobin less than 115 g/L if age ≤ 10 years old; hemoglobin of less than 120 g/L if age > 10 years old
2. High ESR: ESR values greater than 15 mm/h
3. High CRP: CRP greater than 8 mg/L
4. Hypoalbuminemia: albumin less than 35 g/L if age ≤ 10 years old; albumin less than 33 g/L if age > 10 years old
5. High TIBC: TIBC > 80%
6. Iron deficiency: ferritin less than 20 *μ*g/L
7. Low iron levels: serum iron less than 7 *μ*mol/L

### 2.2. Ethics

The proposal of the study was approved by the University of Manitoba Health Research Ethics Board.

### 2.3. Statistics

All data were recorded in an Excel file and imported into STATA version 13 (StataCorp LP, College Station, Texas, USA) for statistical analysis. Continuous variables such as age, mean, median, and SD in those with *H. pylori* were calculated. For categorical data, frequencies and percentages were calculated. The prevalence of colonic changes in those with *H. pylori* was determined.

Two-tailed Student's *t*-test was performed for continuous variables such as age. Fisher's exact test was performed for dichotomous variables such as gender and ethnicity. A *p* value was considered significant if <0.05.

## 3. Results

Approximately 2100 pathology reports of pediatric upper endoscopies were reviewed from January 1996 to May 2015. A total of 231 patients were found to have *H. pylori* gastritis on microscopic examination of biopsy samples obtained from the stomach antrum. Of those, 108 (46.6%) patients were female. The mean age at diagnosis of *H. pylori* gastritis was 12.3 ± 4.1 years. The mean age at diagnosis for females was 12.5 ± 4.2 years and 12.2 ± 4.0 years (*p* = 0.49).

Ethnic background was recorded in 105 of the 231 patients with *H. pylori*. 43.3% of individuals with *H. pylori* were non-White and 2.1% were White. A summary of patients' demographics is provided in Table 1.

Of the 231 patients, 37 patients were found to have ileocolonoscopy performed with upper endoscopy for the following indications:

1. Severe abdominal pain
2. Rectal bleeding
3. Diarrhea of unknown origin
4. Anemia
5. Hypoalbuminemia
6. Abnormal imaging studies such as barium studies

### 3.1. Colonic Changes

Twenty-two (9.5%) patients had abnormal histopathological findings on their colonoscopy biopsy specimens. The mean age at diagnosis of those with colonic changes is 12.1 ± 3.9 years. 8 (36.4%) patients were female with a mean age of 14.5 ± 1.4 years. Fourteen (63.6%) patients were male with a mean age of 10.8 ± 4.3 years (Table 2). The Student *t*-test for comparison of age by gender in those with colonic changes demonstrates males with colonic changes were diagnosed at a significantly earlier age than females with *p* < 0.05 (*p* = 0.03). No significant difference in demographics was found between those who had colonic changes and those who did not have any colonic changes (Table 2). Conditions diagnosed prior to endoscopy for *H. pylori* in this group included celiac disease, inflammatory bowel disease (IBD), and positive FAP gene.  Table 3 summarizes symptoms and laboratory findings for all participants with colonic changes.

**Figure 1:**
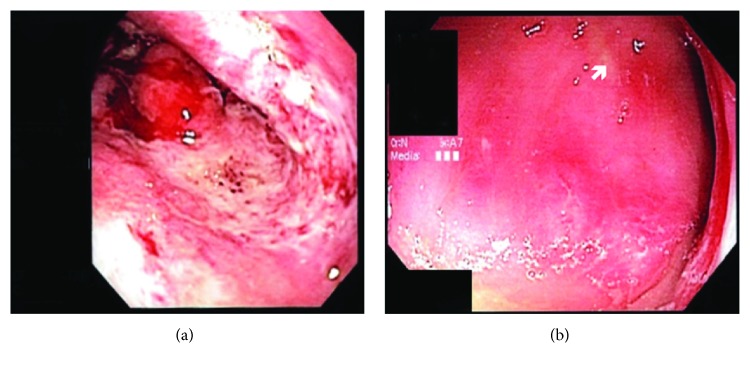
Endoscopy images of patient with necrotic stomach (a). Focal ulceration (denoted by the arrow) and inflammation can be seen on the patient's colonoscopy (b).

Common colonic findings included nonspecific colonic inflammation and colitis (Table 4). There were several patients with unique findings. One patient was found to have a necrotizing gastritis (Figure 1(a)) and acute colitis (Figure 1(b)). This patient was treated with oral anti-*H. pylori* triple therapy (amoxicillin and metronidazole for 2 weeks and omeprazole for 1 month) with significant improvement in his clinical condition and laboratory measures with normalization of his hemoglobin and serum albumin. Six months later, he had a repeated gastroscopy and colonoscopy with biopsies that were completely normal.

**Figure 2:**
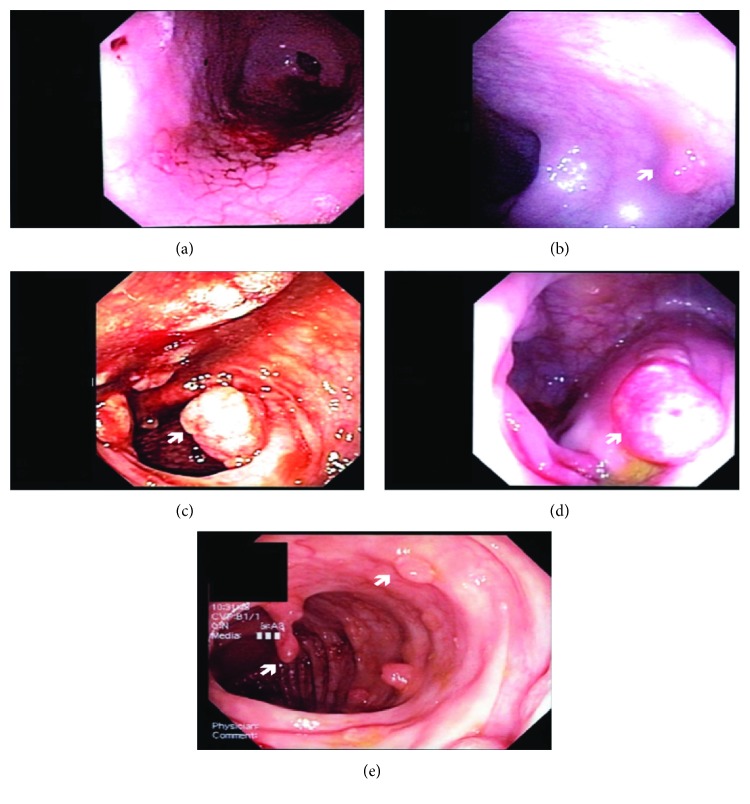
Endoscopy images of patient with antral nodularity (*H. pylori* gastritis) and extensive colonic polyposis (b)–(e).

Another patient was found to have *H. pylori* gastritis (Figure 2(a)) and juvenile colonic polyposis (Figures 2(b)–2(e)) and was thought to be Cronkhite-Canada syndrome but was not confirmed on genetic assessment. He was given oral anti-*H. pylori* triple therapy (amoxicillin and metronidazole for 2 weeks and omeprazole for 1 month). A repeated endoscopy and colonoscopy with biopsies 6 months later confirmed eradication of *H. pylori* gastritis but persistence of colonic polyps; many of them were endoscopically removed and showed hamartomatous changes in histopathology. He and his parents went through further extensive genetic work-up that did not reveal his underlying condition.

Five of 22 patients were also found to have concomitant IBD. They responded well to oral anti-*H. pylori* triple therapy with successful eradication as confirmed on follow-up endoscopies.

### 3.2. No Colonic Changes

Fifteen patients had no significant colonic abnormalities. The mean age of those patients was 11.9 ± 5.1 years. Eight (53.3%) patients were female with a mean age at diagnosis of 10.0 ± 6.0 years. Seven (46.7%) patients were male with a mean age of 14.1 ± 2.9 years. The two-tailed Student *t*-test for comparison of age by gender in those with no colonic changes did not have a significant *p* value (*p* = 0.1). Demographic information for this group can be found in Table 2.  Table 3 summarizes symptoms and laboratory findings in that group. Concomitant medical conditions included vasculitis, asthma, and pancreatic cancer. Variables studied are summarized in Tables 2 and 3.

## 4. Discussion

There have been several studies looking at the adult population with *H. pylori* gastritis and associated colonic changes, but very few are available at present for the pediatric population [11–15]. Our study is novel as our primary aim was to determine the prevalence of colonic changes in pediatric patients with *H. pylori* gastritis and to explore the demographics of this population.

We identified 231 pediatric patients with *H. pylori* gastritis of which 22 (9.5%) were found to have histopathological colonic changes. Between the groups of children with *H. pylori* gastritis with colonic changes and those without colonic changes, those with colonic changes were diagnosed at an earlier age than those without colonic changes. Although we found no significant differences between presenting symptoms in these two groups, those children with colonic changes may have been detected earlier due to the severity of their symptoms or preexisting bowel disease requiring GI referral. Conversely, in females, those without colon changes were diagnosed with *H. pylori* at an earlier age than those with colon changes present. It has been well established in Canada that Aboriginal people and recent immigrants are at a greater risk for acquiring *H. pylori* [5]. Factors such as proper sanitation and increased exposure through contact with endemic regions are likely contributory to the increased risk. We have identified that 50% of those with colon changes are Aboriginal. The data collected exhibits a higher proportion of non-White persons with *H. pylori* with colonic change than those without colonic changes. However, no significant difference was found in the distribution of ethnicities in the groups of colonic change and no colonic change. These findings suggest that although Aboriginal persons and first-generation immigrants are at greater risk for acquiring *H. pylori*, there is no increased risk on having *H. pylori* and colonic changes in these ethnic groups.

The most common symptoms observed in those with colonic change and no colonic changes were abdominal pain, anemia, and weight loss. Specifically, abdominal pain was observed in 54.5% of those with colonic changes and in 20% of those without colonic change. However, there was no significant difference in these proportions between those with colonic changes and those without colonic changes. This implicates that symptoms of *H. pylori* do not tend to follow a different pattern in those with colonic changes and those without colonic changes.

*H. pylori* colonization of the gastric mucosa is virtually always associated with gastritis of predominantly chronic inflammatory cell infiltrates in children [20]. In those with *H. pylori* and colonic changes, 21 patients exhibited chronic active gastritis with *H. pylori* present. The majority of the colonic changes were found to be nonspecific inflammation. These findings implicate that infection with *H. pylori* may have some effect on inflammation in the colon. The pathologic sequelae of *H. pylori* are one which typically follows the chronological order of gastritis, gastric and duodenal ulcers, and gastric lymphoma [21]. Gastric lymphomas are uncommon in children [1]. Given the findings of this study, it is important to consider if colonic inflammation is a part of this pathologic sequelae, specifically in the pediatric population.

One patient who presented with severe abdominal pain and bleeding per rectum had *H. pylori* gastritis and juvenile colonic polyposis and was thought to have Cronkhite-Canada syndrome (CCS) but was not confirmed in genetic assessment. The relationship between colonic polyps and *H. pylori* has not been fully agreed upon in the scientific community. In one study, three patients with *H. pylori* had disappearance of cap polyposis following eradication of *H. pylori* [10]. Associations between *H. pylori* and tubulovillous adenomas and adenocarcinomas [12] have also been shown. However, there are studies which show no relationship between the infection and colorectal cancer or cap polyposis [13, 18]. This patient was further evaluated for CCS. CCS is a rare nongenetic disease with a high mortality rate. The disease presents with characteristic diarrhea, abdominal pain, alopecia, skin hyperpigmentation, and diffuse polyposis throughout the colon. There has been at least one documented case of CCS that has been cured through *H. pylori* therapy [22]. Although our patient did not have complete resolution of colon polyposis following eradication for *H. pylori*, the patient had less colonic polyps in subsequent colonoscopies and experienced reduced abdominal pain and bleeding per rectum. The number of reported cases of CSS worldwide thus far is 450 [23]. An evaluation of a possible association of CCS and *H. pylori* is of significance as highlighted by Kato et al. [22].

Our study found 5 of the 22 patients who had concomitant IBD in conjunction with *H. pylori* infection. The relationship between IBD and *H. pylori* is one of controversy with diverging findings. In one such study, Oliveira et al. isolated and cultured *H. pylori* from the gut and intestinal mucosa of adult patients with Crohn's disease [17]. A study by Sladek et al. has shown the prevalence of *H. pylori* to be lower in pediatric patients with IBD in comparison to patients without IBD [24]. Additional polarity is evident in a meta-analysis and systematic review of literature suggestive of a protective benefit of *H. pylori* against the development of IBD [14], resulting in the conclusion of a prospective inverse relationship between *H. pylori* and IBD. An inverse relationship has also been postulated and demonstrated in a study by Sonnenberg et al. between *H. pylori* gastritis and microscopic colitis, a type of IBD. At present, it is unclear whether our data is representative of these arguments. Further investigations and studies are warranted to explore the relationship of IBD and *H. pylori* in pediatric patients.

Our study is novel but with several limitations. The study is limited by the retrospective design and small sample size for those who had colonoscopy. A major question will be whether our findings are simple association and not cause-effect relationship. [25]

In order to further examine the relationship between *H. pylori* and colonic inflammation, larger, multicenter studies that examine the prevalence of *H. pylori* and colonic inflammation are required in the future. Furthermore, future studies on host factors and environmental factors predisposing one to *H. pylori* infection and colonic inflammation are of interest. Studies pertaining to colonic inflammation and its prevalence in adults with *H. pylori* would allow comparisons to be made to the pediatric population. A cohort study following those with colonic inflammation and *H. pylori* may help to identify if there are any changes in the disease course in these particular individuals.

## 5. Conclusions

A considerable number of pediatric patients with *H. pylori* gastritis in Manitoba had colonic pathologies. Aboriginal population and first-generation immigrants are not at increased risk for having colonic changes in conjunction with *H. pylori.* Our study highlights a possible relationship between *H. pylori* gastritis and colonic changes in children and warrants properly planned prospective studies to confirm our results.

## Figures and Tables

**Table 1:** Demographic data of patients with *H. pylori*.

*H. pylori* (total 231)	Age at diagnosis
Median age (years) ± IQR	14.0 ± 6.0
Mean age (years) ± SD	12.3 ± 4.1
*Ethnicity*	
Aboriginal	85 (36.8%)
Non-Aboriginal	20 (18.7%)
White	5 (2.1%)
Non-White	100 (43.3%)
First-generation immigrant	12 (5.2%)
Unknown	126

**Table 2:** Demographic data of patients with *H. pylori* and colonic changes.

	Colonic change 22 total	No colonic change 15 total	*p* value
Males	13.5 ± 5.0	14.0 ± 7.0	0.04
Females	12.1 ± 3.9	11.9 ± 5.1	
21 boys	11.5 ± 7.0	15.0 ± 2.0	0.03
16 female	15.0 ± 2.0	13.0 ± 11.5	0.06
	14.5 ± 1.4	10.0 ± 6.0	
*Ethnicity*			
Aboriginal	11 (50.0%)	9 (60%)	0.1
Non-Aboriginal	9 (40.9%)	2 (13.3%)	
White	4 (18.2%)	2 (13.3%)	0.6
Non-White	16 (72.7%)	9 (60%)	
First-generation immigrant	5 (22.7%)	0	0.1
Unknown	2	4	

**Table 3:** Symptoms and laboratory data of patients who underwent colonoscopy.

	Colon changes	No colon changes	*p* value
*Symptom*			
Abdominal pain	12 (54.5%)	3 (20%)	0.2
Bleeding per rectum	3 (13.6%)	2 (13.3%)	0.6
Diarrhea	5 (22.7%)	1 (6.7%)	0.5
Syncope	0	0	
Anemia	7 (31.8%)	3 (20%)	0.5
Weight loss	6 (27.3%)	2 (13.3%)	0.6
Unknown	7	8	
Laboratory data			
Low hemoglobin	8	4	0.8
ESR > 15	5	0	
CRP > 8	3	0	
Hypoalbuminemia	4	2	0.8
Iron deficient	4	1	0.9

**Table 4:** Colonic changes found on colonoscopy of 22 patients with colonic change + *H. pylori* infection.

Nature of colonic changes	Number of cases (%)
Colitis	15 (68.1)
(i) Type	
(a) Chronic quiescent	3
(b) Chronic active	4
(c) Acute/active	5
(ii) Severity	
(a) Mild	5
(b) Moderate	5
(c) Severe	2
(iii) Extent	
(a) Focal	3
(b) Diffuse	1
(iv) Others	3
(a) Stricture	1
(b) Granulomatous inflammation	1
(c) Eosinophilic colitis	1
Colonic polyps	5 (22.7)
(i) Juvenile polyp/hamartomatous polyp (Cronkhite-Canada syndrome)	2
(ii) Conventional polyp (tubular adenoma, FAP+)	2
(iii) Hyperplastic polyp	1
Mucosal prolapse/solitary rectal ulcer	2 (9.0)
Total number of cases	22
